# The associations of sitting time and physical activity on total and site-specific cancer incidence: Results from the HUNT study, Norway

**DOI:** 10.1371/journal.pone.0206015

**Published:** 2018-10-23

**Authors:** Vegar Rangul, Erik R. Sund, Paul Jarle Mork, Oluf Dimitri Røe, Adrian Bauman

**Affiliations:** 1 HUNT Research Centre, Department of Public Health and Nursing, Faculty of Medicine, Norwegian University of Science and Technology (NTNU), Levanger, Norway; 2 Nord University, Faculty of Health and Nursing, Levanger, Norway; 3 Cancer Clinic, Levanger Hospital, Nord-Trøndelag Health Trust, Levanger, Norway; 4 Department of Cancer Research and Molecular Medicine, Norwegian University of Science and Technology (NTNU), Trondheim, Norway; 5 Prevention Research Collaboration, School of Public Health, University of Sydney, New South Wales, Sydney, Australia; National Health Research Institutes, TAIWAN

## Abstract

**Background:**

Sedentary behavior is thought to pose different risks to those attributable to physical inactivity. However, few studies have examined the association between physical activity and sitting time with cancer incidence within the same population.

**Methods:**

We followed 38,154 healthy Norwegian adults in the Nord-Trøndelag Health Study (HUNT) for cancer incidence from 1995–97 to 2014. Cox proportional hazards regression was used to estimate risk of site-specific and total cancer incidence by baseline sitting time and physical activity.

**Results:**

During the 16-years follow-up, 4,196 (11%) persons were diagnosed with cancer. We found no evidence that people who had prolonged sitting per day or had low levels of physical activity had an increased risk of total cancer incidence, compared to those who had low sitting time and were physically active. In the multivariate model, sitting ≥8 h/day was associated with 22% (95% CI, 1.05–1.42) higher risk of prostate cancer compared to sitting <8 h/day. Further, men with low physical activity (≤8.3 MET-h/week) had 31% (95% CI, 1.00–1.70) increased risk of colorectal cancer (CRC) and 45% (95% CI, 1.01–2.09) increased risk of lung cancer compared to participants with a high physical activity (>16.6 MET-h/week). The joint effects of physical activity and sitting time the indicated that prolonged sitting time increased the risk of CRC independent of physical activity in men.

**Conclusions:**

Our findings suggest that prolonged sitting and low physical activity are positively associated with colorectal-, prostate- and lung cancer among men. Sitting time and physical activity were not associated with cancer incidence among women. The findings emphasizing the importance of reducing sitting time and increasing physical activity.

## Introduction

There is convincing evidence that regular physical activity (PA) is associated with a reduced risk of several cancers[1]. A recent study with pooled data from 1.44 million adults, found that high leisure-time PA (at the 90th percentile) was associated with reduced risk of 13 of 26 types of cancer examined, with risk reduction of 20% or above for seven of the cancers [2].

Sedentary behaviour (SB) is increasing in the modern society, and appears to differ from physical inactivity; SB is characterized by sitting or lying down, and is defined as any waking behaviour that are done in sitting or reclining posture that expends ≤1.5 metabolic equivalents (METs) [3]. It has been demonstrated that SB may increase the risk of obesity and cardiovascular disease (CVD) mortality, which may be partly independent of PA [4, 5]. Biological mechanisms are still not completely known, but adiposity, metabolic (glucose, insulin), hormonal (sex hormones), inflammatory and vitamin D deficiency among others have been proposed as important factors in the development and progression of some cancers [6]. In epidemiological studies, SB has often been expressed by proxy measures of sitting time, such as television viewing and computer use [7]. A meta-analysis examined SB in relation to cancer risk, but evidence was limited for specific cancer types because of differences in types of SBs and few studies considered specific cancers [8]. A comprehensive prospective study [7] that examined SB in leisure-time in relation to total and specific cancer incidence, found that women who reported leisure-time spent sitting more than 6 h/day had 10% higher risk of total cancer compared to women who reported less than 3 h/day. The association was not modified by PA.

Emerging research indicates that SB is independently associated with cancer; however, few studies have examined the association between PA and sitting time with cancer within the same population [9]. The aim of this study is to explore the relationship and joint associations between PA and sitting time with total and specific cancer incidence, including colorectal, prostate, lung and breast cancer.

## Material and methods

### Study population

**Fig 1:**
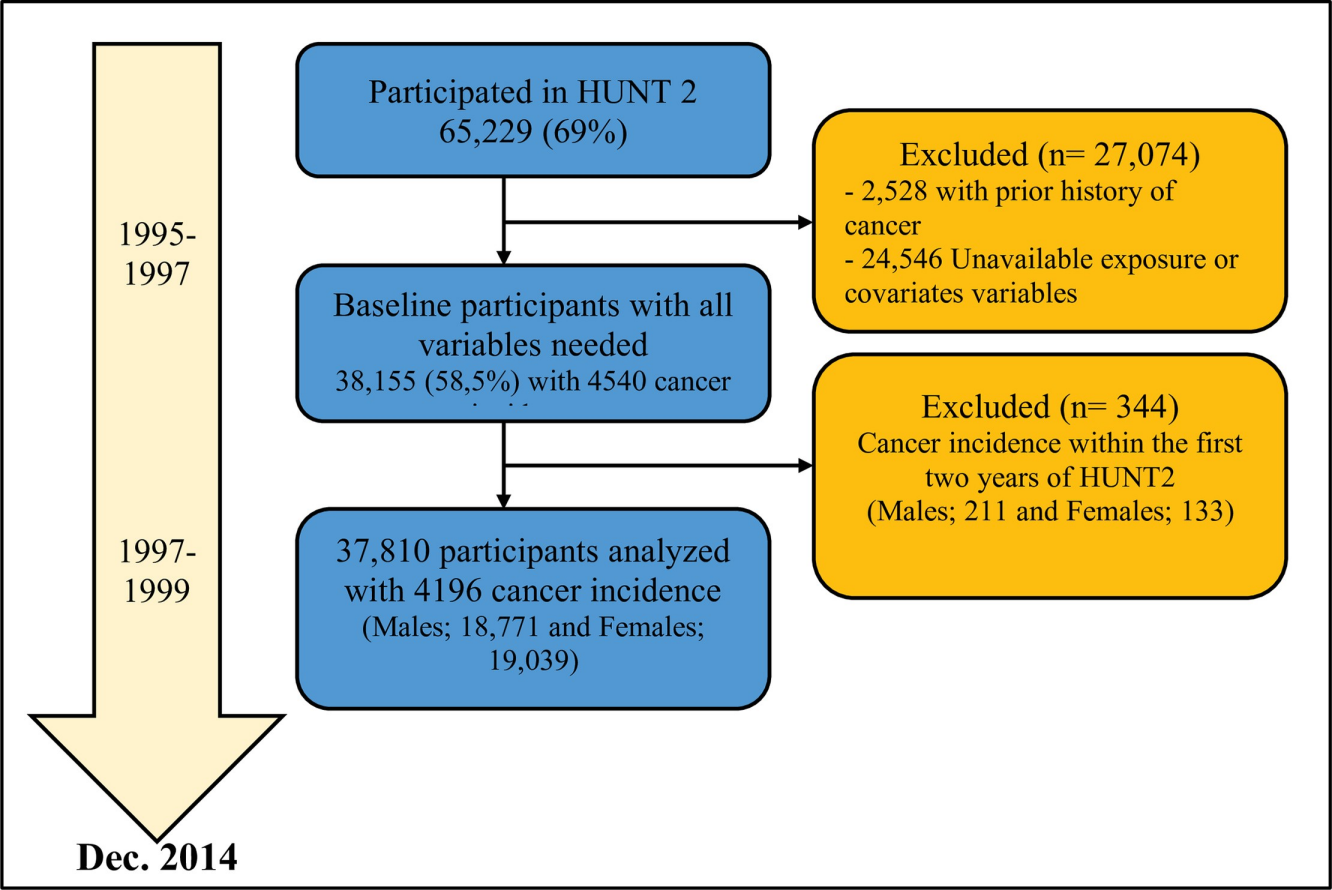
The study flowchart of inclusion over the period.

The Nord-Trøndelag Health Study (HUNT) is a large Norwegian population-based cohort study with three consecutive waves of data collection conducted in 1984–86 (HUNT1), 1995–97 (HUNT2), and 2006–08 (HUNT3). In all three surveys, all residents aged ≥20 years were invited to participate. Detailed information about the HUNT Study is available at: http://www.hunt.ntnu.no/edu/. The present study uses data from the HUNT2 survey, where 93,898 adults were invited and 65,229 (69%) participated. Briefly, information was collected on a range of health and lifestyle-related topics including PA, hours spent sitting, smoking status, alcohol consumption, and educational attainment. The clinical examination included standardised anthropometric measures; body height was set to the nearest centimetre and body weight to the nearest half kilogram. Body mass index (BMI) was calculated as weight divided by the squared value of height (kg/m^2^) and classified into normal weight (<24.9 kg/m^2^), overweight (25.0–29.9 kg/m^2^), obese (30.0–34.9 kg/m^2^) and morbid obese (≥35 kg/m^2^). Very few participants (males, 236; females, 793) were classified as underweight (i.e., <20 kg/m^2^) and none of these were diagnosed with cancer during the follow-up period. Thus, underweight persons were not included in the analysis. For the analysis, the baseline was defined as the first year when information on PA and sitting time was collected (1995). At baseline, we excluded participants with self-reported cancer or diagnosed cancer prior to HUNT2 (from the Cancer Registry of Norway) (2,528), participant with missing covariate information (24,546), and participants with incident cancer within the first two years after baseline (344). The selection of the study participants is shown in Fig 1.

Each participant signed a written consent, and the study was approved by the Regional Committee for Medical and Health Research Ethics (REC), South East, Norway (project no. 2015/663).

### Physical activity and sitting time

PA was assessed by a validated questionnaire [10] that included the following question: “How much of your leisure-time have you been physically active in the last year (Think of a weekly average for the year. Your commute to work counts as leisure-time)?” The participants reported number of hours of either light (no sweating or heavy breathing) and vigorous (sweating and heavy breathing) activity using the response options: “none”, “less than 1 hour”, “1–2 hours”, and “3 hours or more” for each type of activity.

Based on this information, we estimated the metabolic equivalent (MET) hours per week (MET-h/week). The following conversion factors were used when estimating light and vigorous intensity (h/week):”none” = 0 h/week, “less than 1 hour” = 0.5 h/week, “1–2 hour” = 1.5 h/week and “3 hours or more” = 3.5 h/week. Then we assigned the conventionally accepted intensity [11] level for light and vigorous PA, by multiplying hours per week light PA by 2.5 METs and vigorous PA by 7 METs. The resulting continuous variable of MET-h/week were then recoded into three categories based on the recommendations on PA [12], i.e., low (≤8.3 MET-h/week), moderate (8.4–16.6 MET-h/week) and high (>16.6 MET-h/week). The median MET-h/week in each category corresponding to; low PA is about 10 min of moderate intensity activity per day, moderate PA is about 25–35 min of moderate intensity activity per day and high PA is more than 35 min of moderate intensity activity per day. There are some limitations with self-reported methods of physical activity, but as an epidemiological measure it provide information sufficient for a long term cohort exposure concerning participants physical activity patterns [13].

Total sitting time was assessed with a single open-ended question “How many hours do you usually spend sitting down during a 24-hour period?” [14]. Participants were prompted to recall sitting at work, mealtimes, watching TV, sitting in a car, etc. We divided sitting time into two categories, <8 h/day and ≥8 h/day based on meta-analysis on the association between sitting time and PA with all-cause mortality [15].

### Follow up

The unique personal identification number of all Norwegian citizens was used to link each participant’s record in the HUNT Study to information from the Cancer Registry of Norway, Institute of Population-based Cancer Research. Cancer incidence was obtained from 1st of January 1995 to 31st of December 2014, with endpoints of total cancer, and the most common cancers worldwide the last decade: colorectal, prostate, lung and breast cancers. The Cancer Registry receives information mainly from pathology departments, clinicians in both the specialist and primary care, and is considered to be complete. The primary incidence data were coded based on the International Classification of Disease (ICD 10). Cancer incidence was classified using the following ICD-10 codes: total cancer, C00-C97; colorectal cancer (CRC), C18-C21; prostate cancer, C61; lung cancer, C33-34 and breast cancer, C50.

### Statistical analysis

Cox proportional-hazard models were specified to examine the associations of sitting time and PA with total cancer incidence, CRC, prostate cancer, lung cancer and breast cancer using the groups with sitting time <8 h/day and/or PA >16.6 MET-h/week as reference. Time were measured in days from date of participation in HUNT 2 to either; date of emigration, date of death, date of cancer diagnosis or end of follow-up (December 31, 2013) whichever came first. We report Hazard ratios (HR) as effect measures and precision of the estimated associations was assessed by a 95% confidence interval (95% CI)

Sex-specific HRs were estimated for comparability with other studies and to investigate possible sex differences. All estimates were adjusted for the potential confounding effect of age (using age as the time scale in the Cox model). In the fully adjusted models we included potential confounders based on findings in previous studies. These included BMI (<24.9, 25.0–29.9, ≥30 kg/m2), smoking status (never, former or current smoker) alcohol consumption (≥7 units/week, <7 units/week, or abstainer), and educational level primary (primary and lower secondary school), secondary (upper secondary and post-secondary school), and university (first and second stage of tertiary education).

In our combined analysis, we repeated all analysis, but examined the joint association of sitting time and PA with cancer incidence, by directly comparing groups with different amounts of sitting time and PA against those who sat least and those who had highest PA.

All statistical analysis was performed using STATA, version 13.0 software (StataCorp LP, College Station, Texas).

## Results

Baseline characteristics of the study population stratified by sex, sitting time and PA are presented in Table 1. Of the full cohort, 58.5% (n = 38,155) had all variables needed for this study (Fig 1). About 32% (n = 6,103) of males and 25% (n = 4,831) of females reported that they usually spend ≥8 h/day sitting down. Among these, more than 50% reported low PA level (0–8.3 MET-h/week). Participants who spend ≥8 h/day sitting tended to be younger, higher educated and consume more alcohol (Table 1). Participants who reported low PA tended to have lower education compared to participant who reported moderate to high PA.

**Table 1:** Baseline characteristics stratified by sex, sitting time and physical activity (n = 38 155).

		Males					Females				
		Sitting time (h/day)		Physical activity (MET-h/week)			Sitting time (h/day)		Physical activity (MET-h/week)		
		<8 (n = 12,879)	≥8 (n = 6,103)	High (>16.6) (n = 5,619)	Moderate (8.4–16.6) (n = 4,235)	Low (≤8.3) (n = 9,128)	<8 (n = 14,341)	≥8 (n = 4,831)	High (>16.6) (n = 10,166)	Moderate (8.4–16.6) (n = 4,789)	Low (≤8.3) (n = 4,217)
Age, yrs (mean, SD)		48.0 ± 16.0	45.6 ± 13.9	43.3 ± 14.6	50.8 ± 16.4	48.0 ± 14.9	45.0 ± 14.8	42.1 ± 14.4	38.1 ± 12.8	45.5 ± 15.1	46.2 ± 14.6
Sitting time, h/day											
	<8	na	na	3,838 (29.8)	2,962 (23.0)	6,079 (47.2)	na	na	3,065 (21.3)	3,680 (25.7)	7,596 (53.0)
	≥8	na	na	1,781 (29.2)	3,049 (20.9)	3,049 (49.2)	na	na	1,152 (23.9)	1,109 (22.9)	2,570 (53.2)
Physical activity, MET-h/week											
	Low, ≤8.3	6,079 (66.6)	3,049 (33.4)	na	na	na	7,596 (74.7)	2,570 (25.3)	na	na	na
	Moderate, 8.4–16.6	2,262 (69.9)	1,273 (30.1)	na	na	na	3,680 (76.8)	1,109 (23.2)	na	na	na
	High, >16.6	3,838 (68.3)	1,781 (31.7)	na	na	na	3,065 (72.7)	1,152 (27.3)	na	na	na
Education											
	Primary school	3,978 (76.9)	1,194 (23.1)	1,028 (19.9)	1,245 (24.1)	2,899 (56,0)	4,758 (84.5	875 (15.5)	625 (11.1)	1,395 (24.8)	3,613 (64.1)
	Secondary school	7,049 (73.3)	2,571 (26.7)	2,922 (30.4)	2,071 (21.5)	4,627 (48.1)	6,357 (72.2)	2,450 (27.8)	2,070 (23.5)	2,211 (25.1)	4,526 (51.4)
	University	1,852 (44.2)	2,338 (55.9)	1,669 (39.9)	919 (21.9)	1,602 (38.2)	3,226 (68.2)	1,506 (31.8)	1,522 (32.2)	1,183 (25.0)	2,027 (42.8)
BMI, kg/m^2^											
	Normal	4,611 (68.5)	2,122 (31.5)	2,251 (33,4)	1,532 (22.8)	2,950 (43.8)	6,923 (74.0)	2,429 (26.0)	2,440 (26.1)	2,409 (25.8)	4,503 (48.1)
	Overweight	6,509 (67.6)	3,123 (32.4)	2,778 (28.8)	2,187 (22.7)	4,667 (48.5)	5,250 (75.8)	1,676 (24.2)	1,365 (19.7)	1,754 (25.3)	3,807 (55.0)
	Obese	1,534 (67.8)	730 (32.4)	523 (23.1)	463 (20.4)	1,278 (56.5)	1,629 (75.4)	5,32 (24.6)	322 (14.9)	488 (22.6)	1,351 (62.5)
	Morb. Obese	225 (63.7)	128 (36.3)	67 (19.0)	53 (15.0)	233 (66.0)	539 (73.5)	194 (26.5)	90 (12.3)	138 (18.8)	505 (68.9)
Smoking											
	Never	4,843 (65.9)	2,512 (34.1)	2,751 (37.3)	1,476 (20.0)	3,138(42.7)	6,176 (74.2)	2,146 (25.8)	2,156 (25.9)	2,060 (24.8)	4,106 (49.3)
	Former	4,403 (69.5)	1,935 (30.5)	1,651 (26.0)	1,577 (24.9)	3,110 (49.1)	3,528 (74.8)	1,187 (25.2)	995 (21.1)	1,212 (25.7)	2,508 (53.2)
	Current	3,633 (68.7)	1,656 (31.3)	1,227 (23.2)	1,182 (22.3)	2,880 (54.5)	4,637 (75.6)	1,498 (24.4)	1,066 (17.4)	1,517 (24.7)	3,552 (57.9)
Alcohol use, units per week											
	No alcohol	2,512 (75.6)	812 (24.4)	750 (22.6)	884 (26.6)	1,690 (50.8)	4,603 (78.8)	1,236 (21.2)	872 (14.9)	1,475 (25.3)	3,492 (59.8)
	< 7 last week	9,016 (67.5)	4,348 (32.5)	4,035 (30.2)	2,886 (21.6)	6,443 (48.2)	9,315 (73.4)	3,373 (26.6)	3,146 (24.8)	3,131 (24.7)	6,411 (50.5)
	≥ 7 last week	1,351 (58.9)	943 (41.1)	834 (36.3)	465 (20.3)	995 (43.4)	423 (65.6)	222 (34.4)	199 (30.8)	183 (28.4)	263 (40.8)

Values are expressed as mean ± standard deviation or number (%)

During a median follow-up of 16 years (608,445 person years), 4,196 persons were diagnosed with cancer (excluding incidence within the first two years after HUNT2) among the analytical sample. Of these, 643 were diagnosed with CRC, 391 with lung cancer, 889 with prostate cancer (men), and 513 with breast cancer (women). The other major contributors for total cancer incidence was leukemia, melanoma, urinary tract, ovarian and central nervous system cancers. Table 2 presents the HRs for the associations between sitting time, PA and total and specific cancer incidence for men and women separately.

**Table 2:** Hazard ratios for associations between sitting time, physical activity and total and specific cancer incidence.

				Sitting time (h/day)			Physical activity (MET-h/week)			
			Events/person-yrs	<8	≥8	p-value	High (>16.6)	Moderate (8.4–16.6)	Low (≤8.3)	p-value for trend
Total cancer										
	Age-adjusted									
		Men	2435/295342	1.00 (ref)	1.07 (0.98, 1.18)	0.11	1.00 (ref)	1.01 (0.90, 1.13)	1.08 (0.98, 1.20)	0.12
		Women	1761/312734	1.00 (ref)	1.05 (0.94, 1.17)	0.42	1.00 (ref)	1.00 (0.86, 1.16)	0.95 (0.83, 1.08)	0.42
	Multivariate^1^									
		Men	2435/295342	1.00 (ref)	1.08 (0.98, 1.18)	0.11	1.00 (ref)	1.00 (0.89, 1.12)	1.06 (0.95, 1.17)	0.30
		Women	1761/312734	1.00 (ref)	1.02 (0.91, 1.14)	0.78	1.00 (ref)	1.00 (0.86, 1.16)	0.95 (0.83, 1.09)	0.47
Colorectal cancer										
	Age-adjusted									
		Men	388/305186	1.00 (ref)	1.16 (0.93, 1.45)	0.18	1.00 (ref)	1.23 (0.92, 1.65)	1.29 (1.00, 1.68)	0.05
		Women	255/321504	1.00 (ref)	1.00 (0.74, 1.36)	0.99	1.00 (ref)	1.05 (0.72, 1.53)	0.83 (0.58, 1.18)	0.29
	Multivariate^1^									
		Men	388/305186	1.00 (ref)	1.14 (0.91, 1.43)	0.26	1.00 (ref)	1.26 (0.94, 1.69)	1.31 (1.00, 1.70)	0.05
		Women	255/321504	1.00 (ref)	1.02 (0.75, 1.39)	0.91	1.00 (ref)	1.05 (0.72, 1.54)	0.82 (0.57, 1.18)	0.29
Prostate cancer (men)										
	Age-adjusted		889/304927	1.00 (ref)	1.32 (1.14, 1.53)	0.00	1.00 (ref)	0.78 (0.65, 0.93)	0.94 (0.80, 1.11)	0.47
	Multivariate^1^		889/304927	1.00 (ref)	1.22 (1.05, 1.42)	0.01	1.00 (ref)	0.83 (0.68, 0.99)	1.01 (0.86, 1.19)	0.87
Lung cancer										
	Age-adjusted									
		Men	242/306817	1.00 (ref)	1.02 (0.76, 1.36)	0.90	1.00 (ref)	1.54 (1.03, 2.29)	1.82 (1.27, 2.61)	0.00
		Women	149/322643	1.00 (ref)	0.82 (0.53, 1.25)	0.35	1.00 (ref)	0.93 (0.52, 1.64)	1.22 (0.74, 2.03)	0.44
	Multivariate^1^									
		Men	242/306817	1.00 (ref)	1.14 (0.86, 1.54)	0.36	1.00 (ref)	1.27 (0.85, 1.90)	1.45 (1.01, 2.09)	0.04
		Women	149/322643	1.00 (ref)	0.85 (0.55, 1.31)	0.46	1.00 (ref)	0.77 (0.44, 1.38)	0.95 (0.57, 1.59)	0.84
Breast cancer (women)										
	Age-adjusted		513/319365	1.00 (ref)	1.12 (0.91, 1.36)	0.28	1.00 (ref)	0.89 (0.68, 1.16)	0.86 (0.68, 1.09)	0.21
	Multivariate^1^		513/319365	1.00 (ref)	1.04 (0.85, 1.27)	0.74	1.00 (ref)	0.92 (0.70, 1.19)	0.93 (0.73, 1.17)	0.52

^1^ Adjusted for age, education, smoking, alcohol, BMI

Men who reported sitting for ≥8 h/day had a multi-adjusted hazard ratios (HR) of 1.14 (95% CI 0.91, 1.43) for CRC compared to the reference category of men sitting <8h/day. For PA, the multi-adjusted analyses showed that low PA among men was associated with increased risk of CRC (HR 1.31, 95% CI 1.00, 1.70) and lung cancer (HR 1.45, 95% CI 1.01, 2.09) compared to the reference category of high PA. Further, we observed that men who reported moderate PA had reduced risk of prostate cancer (HR 0.83, 95% CI 0.68, 0.99) compared to the reference category of high PA. There was no association between PA or sitting time with cancer incidence among women.

The partially adjusted HR for prostate cancer incidence associated with sitting time showed a significant association between sitting 8 hours or more per day, compared those sitting less than 8 hours per day (HR 1.32, 95% CI 1.14, 1.53). When adjusting for education, smoking, alcohol and BMI, those who reported sitting 8 hours or more per day had an 22% (95% CI 1.05, 1.42) higher risk for prostate cancer, compared those sitting <8 hours per day. Moderate PA between 8.4–16.6 MET-h/week was significantly associated with decreased prostate cancer incidence (HR 0.83, 95% CI 0.68, 0.99), compared with high PA level (Table 2).

For men who had a low PA level (≤8.3 MET-hr/week) had a 45% (95% CI 1.01, 2.09) higher risk for lung cancer, compared those who had high PA level.

**Fig 2:**
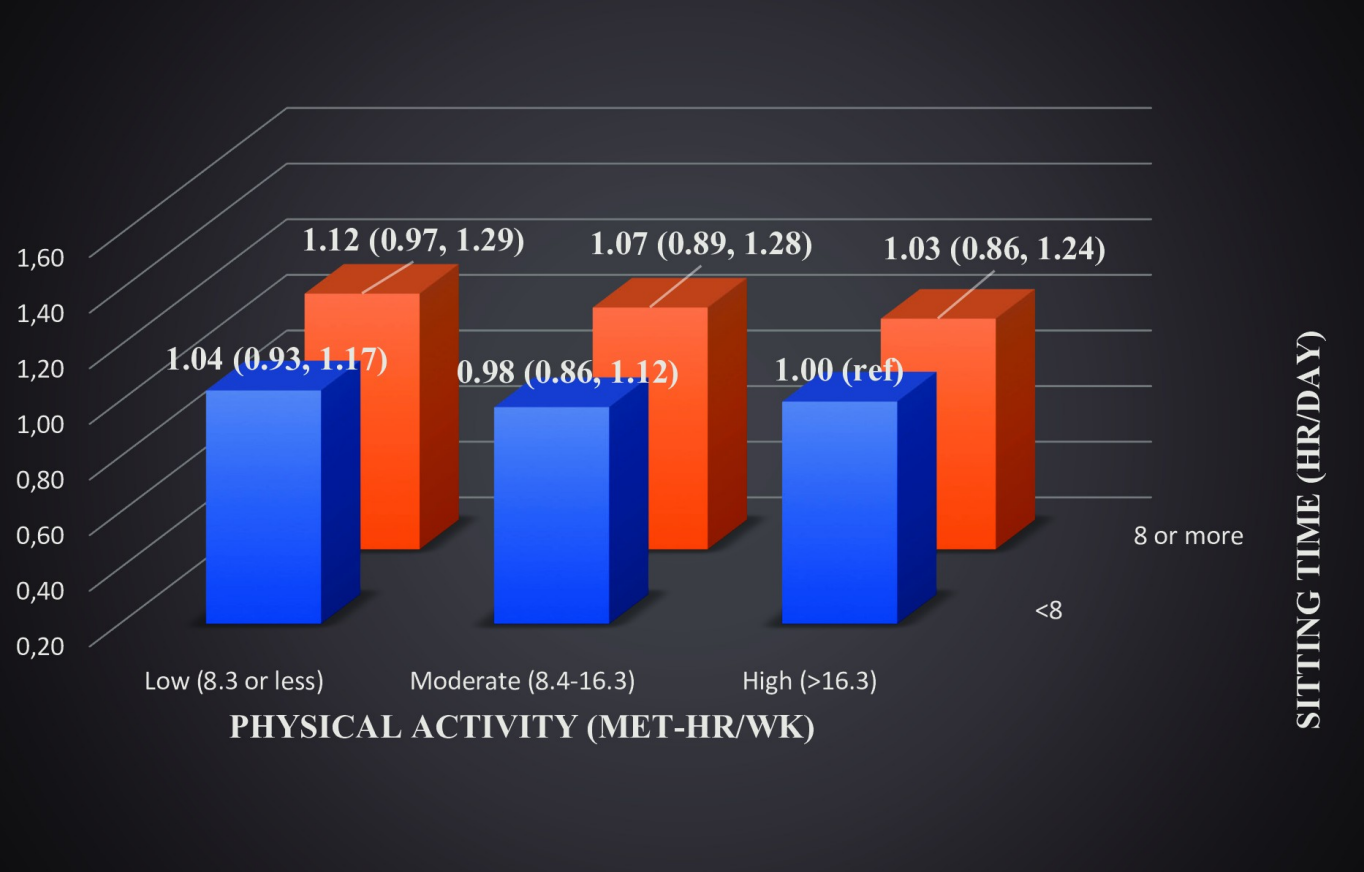
Joint association of sitting time and physical activity with total cancer, adjusted HRs in men.

When the joint association of sitting time and PA was examined for total cancer incidence, we found (age adjusted model) that men who sat ≥8 hours per day and reported a low physical active level had an 16% (95% CI 1.02, 1.34) increased risk compared with men who sat less and were more physically active (>16.6 MET-hr/week) (S1 Table). In the fully adjusted model (Fig 2), the risk was slightly lower and not significant (HR 1.12, 95% CI 0.97, 1.29) but showed a dose-response relationship.

**Fig 3:**
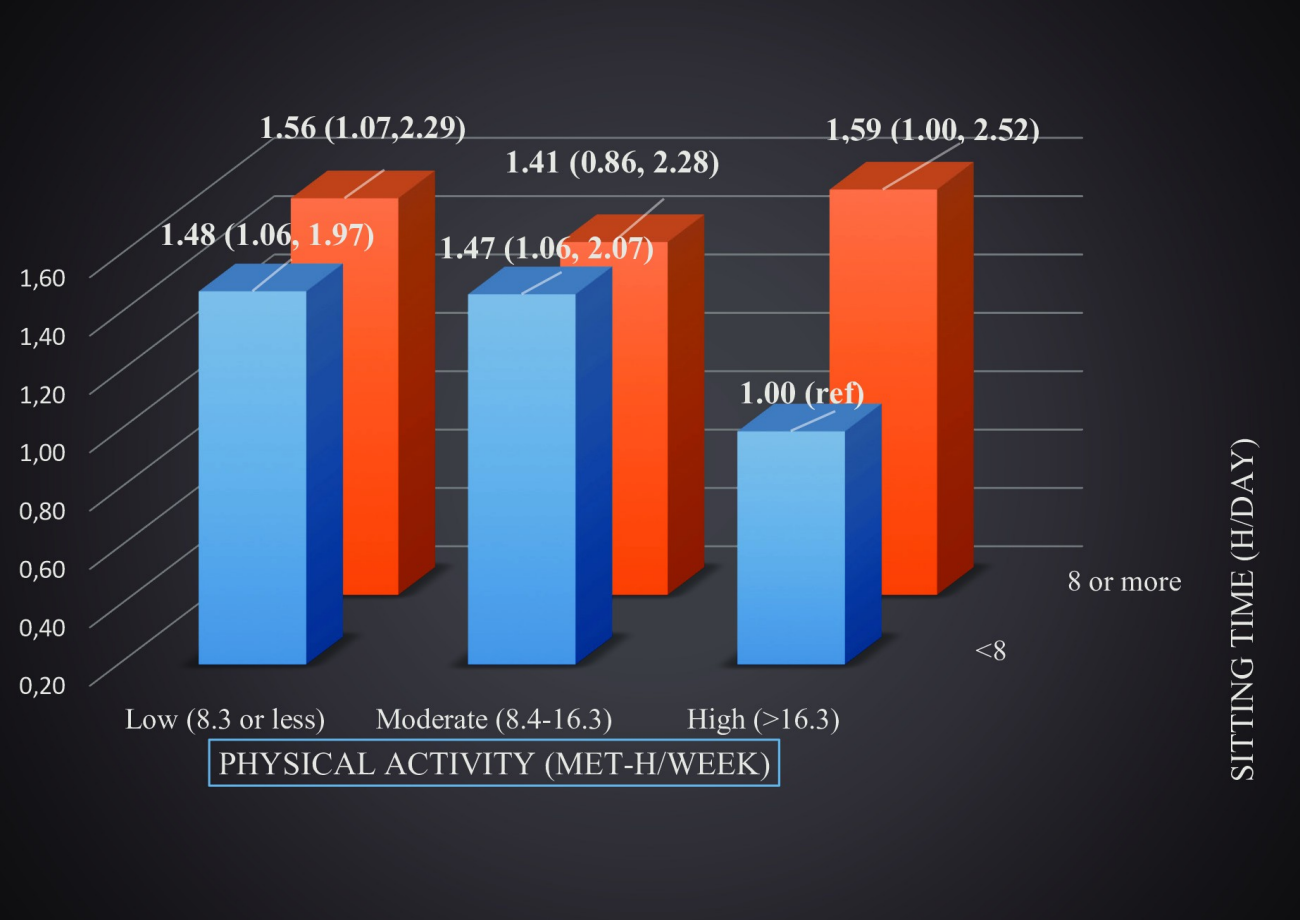
Joint association of sitting time and physical activity with colorectal cancer, adjusted HRs in men.

In the joint analysis of sitting and PA, men who were sitting for ≥8 h/day had 41–59% increased risk of CRC regardless of PA level compared to men sitting for <8h/day and who were highly physically active (Fig 3).

For women, only those with high PA level (>16.3 MET-hr/week) and reported ≥8 h/day of sitting time had an increased risk for CRC (HR 2.12, 95% CI 1.10, 4.08) compared women sitting <8 h/day (S2 Table).

The full sample analysis (not excluding incident cases within two years of HUNT2) showed little change in effect size and statistical significance from the sensitivity analysis except the joint associations of sitting and PA with CRC incidence in men, whereas HRs for high PA and high sitting became not significant (HR 1.43, 95% CI 0.91, 2.25).

## Discussion

The main findings in this longitudinal population-based study were that prolonged sitting time is independently associated with increased risk of prostate cancer while moderate to high leisure-time PA may reduce the risk of specific cancer types, particularly colorectal and lung cancer. These associations were not observed among women. An unexpected finding was that moderate leisure-time PA was associated with reduced risk of prostate cancer compared to a high PA level. For the analyses of joint associations, we observed that prolonged sitting time was associated with ~40–60% increased risk of CRC regardless of level of leisure-time PA. For total cancer, we observed no effect of sitting within the different levels of leisure-time PA. These data are one of the first prospective studies investigating the association between leisure-time PA and daily sitting time with total and specific cancer incidence within the same population.

Several studies have examined sedentary behavior using proxy measures of sitting time such as TV-viewing time in relation to cancer risk [7, 16–19]. These studies reported an association between sedentary behavior and breast, ovarian cancers, multiple myeloma, colon- and lung cancer risk. A recent systematic review and meta-analysis, including prospective cohorts and 22 case-control studies, found an association between prolonged sedentary time and increased risk of colon-, endometrial- and lung cancer [8]. Similar evidence was found in another meta-analysis that included 17 prospective studies [20]. In this latter study, sedentary behavior was associated with increased risk of colorectal, lung, breast and endometrial cancers.

In the present study, only prostate cancer risk was independently increased (22%, p = 0.01) associated with sitting more than 8 hours per day. Further, we noted that moderate PA significantly decreased the risk. Similar results were shown in a large cohort study in Sweden where over 45,000 men aged 49–75 were followed for nine years, where men who mostly sit during their main work or occupation, had a 20% higher risk than men who sit half of the time. They found that the rate ratio linearly decreased by 7% for total, 8% for localized and 12% for advanced prostate cancer for every 30 min per day increment of lifetime walking or bicycling in the range of 30 to 120 min per day [21]. The authors also hypothesized that the biological mechanisms by which PA may decrease prostate cancer risk could be through certain hormones associated with prostate carcinogenesis, including insulin resistance [22], adiponectin levels [23, 24], insulin-like growth factors [25] and testosterone [26]. More recently, muscle- and fat-related proteins as follistatin, myostatin, activin and inhibin has been shown to facilitate proliferation, dissemination, inhibition or apoptosis of human prostate cancer cells as reviewed by Wekesa et al. [27]. Despite lacking evidence on the effect of PA on these proteins in prostate cancer patients, studies carried out in healthy subjects showed that PA increases plasma follistatin, decreases circulating inhibin and activin, decreases tissue/circulating myostatin and downregulates the expression of activin receptor, thus the opposite could infer a plausible mechanism of action for inducing prostate cancer.

An unexpected finding was that moderate leisure-time PA was associated with reduced risk of prostate cancer compared to high PA. However, this was similar to the findings of a large recent study were vigorous leisure-time PA was associated with an increased risk (2). In one older study of our population, high leisure-time PA seemed to protect against aggressive cancer but was not associated to prostate cancer in general [27, 28].

Some studies have shown that prostate cancer may be more common in advantaged social groups [29]. Lund Nilsen et al. found that men with high socio-economic status (i.e. long/high education) had an elevated risk of prostate cancer [30]. However, the reason why prostate cancer incidence is higher among men with high education remains unknown. In our study, we found that 55.9% of men with high education level (University) were sitting for ≥8 h/day, while among those with low educational level only 23.1% were sitting for ≥8 h/day. Several mechanisms could explain the observed association. When a substantial amount of time is spent sitting, this could mean less PA and it may lead to higher energy intake, resulting in increased risk of overweight/obesity. However, we observed that the association between sitting time and prostate cancer remained also after adjusting for BMI and education. This contrasts with findings reported by two other prospective studies where sitting time was not associated with prostate cancer incidence risk [7, 17]. Thus, to clarify the exact nature and mechanism of the association between prolonged sitting and prostate cancer warrants further investigation.

In our study, low leisure-time PA showed an adjusted 31% (p = 0.05) increased risk of CRC in men, an association that has been well established previously [31, 32]. A meta-analysis performed by the continuous update project (CUP) of the World Cancer Research Fund, showed that total PA was associated with reduced risk of CRC [33]. In a large Swedish study of men, the leisure-time PA was protective, while occupational activity was not, in line with our findings [34]. Concerning gender differences, where we found associations among men, but not in women. Similarly, CUP concluded that the effect was strong and consistent in men, but less strong in women.

Biological mechanisms involved are still unknown, but all the above mentioned are plausible factors. However, recent data point to epigenetic factors, that is DNA modifications that do not alter the nucleotide sequence but still influence gene expression and may also be heritable as important factors specifically for colorectal neoplasia. These mechanisms include modifications of histones, non-coding RNA such as microRNA (miRNA) expressions, and DNA methylation variations [35].

Lung cancer is the most common cancer in men worldwide, with an annual incidence of about 1.2 million cases globally, (16.7% of the total cancer incidence) where tobacco smoking is the main cause [36]. In women, the incidence rates are generally lower, with highest rates in Northern America and Northern Europe. In our study, men with low leisure-time PA had a 45% (p = 0.04) increased risk for lung cancer incidence, adjusted for age, education, smoking, alcohol and BMI. This is similar with two recent meta-analyses, showing that leisure-time PA significantly reduce the risks for lung cancer in both men and women. Moreover, one meta-analysis indicates that PA lowers risk of lung cancer among smokers, that indicate a protective effect of PA against the deleterious effects of smoking [37].

The biological mechanisms of the preventive effect of PA on lung cancer are probably highly complex, and several mechanisms are investigated, among them pulmonary function improvement, reduction of carcinogenic agents in the lungs, a better DNA repair capacity, immune function enhancement, reduced inflammation, changes in growth factor levels and possible gene-environment interactions [38–40].

Our joint analysis of PA and sitting time identified one main finding. Among those sitting for ≥8 h/day the risk for CRC was similar across categories of PA level. This suggests that prolonged sitting increase the risk of CRC may be independent of PA. This is similar to what Cong et al. [41] found in a meta-analysis of observational studies of the associations of SBs with colon and rectal cancer, i.e., a 30% increased risk for colon cancer and 34% for rectal cancer after adjustment for PA. In line with this, another meta-analysis of prospective studies found that prolonged SB was independently associated with increased risk of incident colorectal, breast and lung cancers [20]. Concerning the joint relationship with other site-specific cancers, the evidence remains unclear. We need further evidence of the combined association between sitting time and PA, as well as studies of biological mechanisms with cancer incidence to understand in more detail how increased PA and reducing total sitting time may decrease cancer risk.

An important question is why there was no significant association of PA/SB with total cancer, site-specific cancer, not even breast cancer in women, since this has been found in several previous studies and meta-analyses [7]. We believe that there are two possible factors. The baseline survey here was in 1995–97 and since then, in Norwegian society, there has been an increased focus on diet, PA and weight control, especially among women. Since the follow-up time is 16 years, many women may have changed their life-style during these years and thus changed their risk profile [42]. The other factor is the introduction of the National mammography screening starting in 2004, increased the incidence and the rate of early tumours in younger women, including even indolent types resulting in over diagnosis [43].

Consequently, as the age of the women diagnosed is younger, the effects of SB reached its full impact and other factors, as genetically, hormonal supplements and menarche and parity plays a stronger role. Finding indolent tumours classified as cancer also induce an important bias in this situation [44].

The strengths of our study include the population-based prospective design with 16-years of follow-up, the large sample size, and the availability of several relevant covariates as well as the completeness and quality of the Norwegian Cancer Registry that provides accurate diagnoses. These features reduce the potential risk of recall, measurement and selection biases, and may increase the generalizability of these findings. Prostate-specific antigen (PSA) testing may be considered to introduce bias, as this screening technique may only detect low grade tumors with no clinical relevance. However, in Norway there is no official recommendation on PSA testing for screening purposes in men without symptoms, and thus the potential bias that could have been introduced by PSA is of lesser importance. Additionally, this is one of the first studies to investigate the associations between PA and sitting time with several site-specific cancers within the same population, thereby enabling us to assess the independent associations between sitting time and PA.

Limitations include the fact that sitting time and PA were self-reported. However, it is worth nothing that the questions on leisure-time PA have been shown to have acceptable validity, especially for high PA [10]. Selection bias may be higher in surveys with high participation rates than those with lower participation rates [45], as the differences between participants and nonparticipants may exaggerate real differences between participants and the eligible population sampled. Compared to nonparticipation questionnaire and records from general practice it seems that the present study to be somewhat underestimated for chronic diseases and higher prevalence among nonparticipants of cardiovascular diseases, diabetes and mental distress [46]. Another limitation is the single baseline measurement, and we could not assess if changes in PA and/or sitting time over time may have influenced the results. The mean age at baseline among men was 47 years and women 44 years and thus their reporting may be representative of their adult life-time PA and sitting pattern. Finally, lack of a covariate variable associated with diet is considered as a limitation in this study.

## Conclusions

In conclusion, prolonged sitting time and low leisure time PA are associated with incidence of some specific types of cancer in men, including colorectal, prostate and lung cancer. Specifically, prolonged sitting is associated with increased risk of prostate cancer while low leisure time PA is associated with increased risk of colorectal- and lung cancer. Moderate leisure time PA is associates with reduced risk of prostate cancer. Our results reaffirm PA as a key component of population-wide cancer prevention, and we need to consider recommendations about decreasing time spent sitting.

## Supporting information

S1 Table Joint associations of sitting and PA with total cancer—crude estimates in men. (PDF)

S2 Table Joint associations of sitting and PA with colorectal cancer–adjusted estimates in women. (PDF)
