# Supplementary material for: The associations of sitting time and physical activity on total and site-specific cancer incidence: Results from the HUNT study, Norway
Source: PLoS One. 2018 Oct 23;13(10):e0206015. doi: 10.1371/journal.pone.0206015 (PMC6198967; doi:10.1371/journal.pone.0206015)
Supplement: S1 Table — (PDF) [file pone.0206015.s001.pdf]

Supporting information

S1 Table. Joint associations of sitting and PA with total cancer - crude estimates in men (n=18 771)

MET-h/week	Sitting time (h/day)	
	Low <8	High ≥8
Low (≤8.3)	**1.07 (0.96, 1.20)**	**1.16 (1.02, 1.34)**
Moderate (8.4-16.3)	**1.02 (0.90, 1.16)**	**1.10 (0.92, 1.31)**
High (>16.3)	**1.00 (ref)**	**1.02 (0.86, 1.22)**

adjusted for sex and age
