# Supplementary material for: The associations of sitting time and physical activity on total and site-specific cancer incidence: Results from the HUNT study, Norway
Source: PLoS One. 2018 Oct 23;13(10):e0206015. doi: 10.1371/journal.pone.0206015 (PMC6198967; doi:10.1371/journal.pone.0206015)
Supplement: S2 Table — (PDF) [file pone.0206015.s002.pdf]

Supporting information

S2 Table. Joint associations of sitting and PA with colorectal cancer – adjusted estimates in women (n=19 149)

MET-h/week	Sitting time (h/day)	
	Low <8	High ≥8
Low (≤8.3)	**1.04 (0.49, 1.48)**	**0.85 (0.49, 1.48)**
Moderate (8.4-16.3)	**1.20 (0.76, 1.91)**	**1.19 (0.62, 2.29)**
High (>16.3)	**1.00 (ref)**	**2.12 (1.10, 4.08)**

Age, sex, education, smoking, alcohol, BMI
